# Gadolinium-doped fluorescent carbon quantum dots as MRI contrast agents and fluorescent probes

**DOI:** 10.1038/s41598-022-22518-0

**Published:** 2022-10-21

**Authors:** Mohammad Jafar Molaei

**Affiliations:** Faculty of Chemical and Materials Engineering, Shahrood University of Technology, Shahrood, Iran

**Keywords:** Imaging, Nanobiotechnology

## Abstract

In this research passivated gadolinium-doped carbon quantum dots (Gd-doped CQDs) were synthesized from starch by a hydrothermal method. The X-ray diffraction (XRD) pattern of the Gd-doped CQDs showed the formation of highly amorphous carbon. The Fourier transform infrared spectroscopy (FTIR) results suggested that the CQDs are functionalized with C-N and N–H bonds. The synthesized CQDs with a size distribution of 2–8 nm have an absorption peak at 271 nm in UV–Visible spectroscopy (UV–Vis). The photoluminescence (PL) in CQDs was dependent on the excitation wavelength. The QY of the synthesized CQDs was calculated to be 13.2%. The Gd-doped CQDs exhibited sustained PL in ionic solutions with different ionic strengths and different temperatures up to 65 °C. Fluorescence imaging on mouse C_34_/connective tissue-L929 cells confirmed that Gd-doped CQDs could be well distributed over the cytoplasm. The magnetic resonance imaging (MRI) showed that the Gd-doped CQDs have extremely high longitudinal and transverse relaxivity values of as high as 218.28 mM^−1^ s^−1^ and 364.68 mM^−1^ s^−1^. The synthesized Gd-doped CQDs are promising candidates as multifunctional imaging probes and MRI contrast agents in biomedical diagnosis and brain mapping applications.

## Introduction

Fluorescence imaging and magnetic resonance imaging (MRI), categorized as multimodal imaging, are exceptionally beneficial in biomedical technology since it provides simultaneous highly sensitive functional imaging and high-resolution histological information^1^. Contrast agents have been applied to improve the obtained information from the MRI^2^. The contrast agents for MRI can be categorized under two types: the T1 and T2 contrast agents. Superparamagnetic iron oxide nanoparticles (SPIONs) are one of the contrast agents that are used for the T2 mode. During recent decades, much attention has been devoted to the T1 MRI contrast agents, since the T2 mode of MRI can be confused with hypointense lesions and the blooming effect generated by SPIONs^3^. Different T1 contrast agents based on the Gd^3+^ are gadolinium doped iron oxide^4^, gadolinium PGMA-based supramolecular polycations (PGEDGd@PGEAs)^5^, gadolinium oxide nanoparticles (Gd_2_O_3_)^6^, and gadolinium functionalized carbon materials (graphene oxide^7^, carbon nanotube^8^, fullerene^9^). However, most of the mentioned T1 contrast agents are single-modal only for MRI^3^. Contrast agents with semiconductor quantum dots (QDs) and gadolinium(III) (Gd(III)) chelates have received great interest in recent years^10,11^. Gd(III) exhibits bright contrast in longitudinal relaxation rate (r_1_) and is clinically used widely after lowering its cytotoxicity^1^.

An ideal probe for dual-modal fluorescence/MR imaging should provide high relaxivity and enhanced fluorescence intensity while should show proper stability in the body and should consist of low toxicity materials. CQDs can be used as multi-modal nanoprobes due to their inherent fluorescence emission, water-solubility, conserving their optical properties in biological environments, and low toxicity^12^.

CQDs are surface passivated and functionalized carbon nanoparticles that have fluorescence properties. These nanostructures are water-soluble, non-blinking, and have a high cross-section for two-photon excitation. These characteristics along with nontoxicity have made CQDs potential nano-probes for bioimaging^13^. The CQDs have sizes almost below 10 nm. They could be amorphous or crystalline with carbon atoms having sp^2^ hybridization^14^. Oxygen-containing functional groups (hydroxyl and carboxyl) that have surrounded the CQD’s surface make them water-soluble. These functional groups on the surface of the CQDs form stable colloids in polar organic or aqueous solvents which is beneficial in biomedical applications, in comparison with graphene quantum dots that miss having good water solubility^15^. The CQDs can emit PL in the near-infrared (NIR) by excitation in the NIR region. This is advantageous in bioimaging, drug delivery, cancer therapy, and photoacoustic imaging. The PL has a wide range in CQDs from deep ultraviolet to NIR. The PL emission could be tuned in CQDs by controlling size, altering surface and edges, and doping heteroatoms^16^.

The fluorescence of the CQDs can be originated from two different mechanisms that the first class is bandgap transitions aroused by conjugated π-domains. The π-domains in CQDs are isolated by forming sp^2^ hybridized islands. For fluorescence emission, there should not be connections between sp^2^ islands. The sp^2^ islands' connections lead to fluorescence quenching. Light absorption by π-electrons in the sp^2^ islands forms excitonic states. The second class of mechanisms that can be the reason for fluorescence emission originates from surface defects. The sites with non-perfect sp^2^ domains lead to surface energy traps^17^.

Different chemical and physical routes have been applied to produce CQDs including laser ablation^18^, thermolysis^19^, electrochemical^20^, ultrasonic^21^, microwave aided synthesis^22^, and hydrothermal synthesis^23–25^. Among the mentioned methods for the synthesis of CQDs, the hydrothermal method has the advantages of synthesizing CQDs with low toxicity, biocompatibility, superior PL performance, chemical, and thermal stability, and low photo-bleaching^26^. CQDs have been synthesized from different natural sources such as orange peel^27^, banana peel^25^, coffee ground^28^, lemon juice^29^, etc. Synthesis of CQDs from natural sources might result in a product with a decreased toxicity in comparison to other chemicals as the starting material^15^.

The fluorescence emission in CQDs originates from isolated sp^2^ carbon clusters and defect sites inside the carbon matrix. A higher ratio of sp^3^ carbon atoms and heteroatoms like phosphorus, sulfur, nitrogen, and oxygen induces more defect sites in the sp^2^ carbon clusters which results in an improvement in the fluorescence emission^30^. For example, CQDs synthesized from a vitamin B1 carbonization method have enhanced fluorescence emission due to the incorporation of heteroatoms and phosphorus into the CQDs^31^. Surface functional groups and surface oxidation of CQDs have a considerable effect on their fluorescence emission as well^32^. Functional groups such as C = N, C = O, C = S, etc. introduce different energy levels into the CQD which result in fluorescence modulation^33^. Functional groups have an essential effect on the performance of the CQDs in different applications. For example, for the CQDs synthesized from triphenylphosphonium (TPP) as selective tetracycline sensors and vehicle for mitochondria labeling in cancer cells, the TPP residues on the surfaces of CQDs could easily recognize and target mitochondria in cancer cells^34^.

Gadovist® is gadolinium- (Gd)-based contrast agent that can be utilized for the whole body. This contrast agent is used for the visualization and detection of the areas with disruption of the blood–brain barrier (BBB) and/or abnormal vascularity in MRI of the spine, brain, etc.

When Gadovist® as a macrocyclic paramagnetic molecule is placed in a magnetic field, magnetic moments develop. The enhancement of the relaxation rate of water protons in the vicinity of the magnetic field results in the brightness of the tissues^35^.

Different Gd-doped CQDs have been used for multifunctional bioimaging and MRI contrast agents^36–40^. Also, numerous doped QDs have been developed for bimodal imaging, for example using Mn or Fe as dopants^41–43^. However, most of the investigated contrast agents are synthesized from chemical precursors that could be toxic to human body cells. In this research, starch as a natural polymeric carbohydrate for the production of CQDs via hydrothermal route was used. Hydrothermal conditions applied to the starch can result in the carbonization of this natural polymer and the production of CQDs that can be collected from the residue. Starch, polyethyleneimine, and Gadovist® were used to synthesize Gd-doped CQDs. Polyethyleneimine was used as passivating and N-doping agent. The potential of the CQDs for fluorescence bioimaging and contrast agents for MRI was investigated.

## Experimental procedure

### Chemicals and materials

Starch soluble was purchased from Merck. Polyethyleneimine was provided from Sigma-Aldrich. Gadovist® which is a gadolinium-based MRI contrast agent was provided by Cobel Darou. RPMI-1640 medium, glutaraldehyde, Fetal bovine serum (FBS), trypsin–EDTA, and penicillin/streptomycin were obtained from GIBCO.

### Synthesis of Gd-doped CQDs

The CQDs were synthesized through the hydrothermal method. Starch as the carbon source, polyethyleneimine as the surface passivation agent, and Gadovist® for the doping of Gd^3+^ into the CQDs were used as the starting materials. For the synthesis of the CQDs, 4 g of starch.

was suspended in 80 mL of deionized water and the mixture was stirred for 2 h at 80 °C until complete solvation of starch in deionized water. Then 100 μL polyethyleneimine and 2 mL of Gadovist® (0.5 mmol/mL) were added to the suspension and stirring continued for one more hour at room temperature. The solution afterward, was transferred into a Teflon-lined stainless steel autoclave. The autoclave was put into an oven and heated up to 180 °C and stayed for 8 h.

The autoclave was cooled down in the oven and the solution was turned brown after the hydrothermal process. The product of hydrothermal synthesis was centrifuged for 20 min at 4000 rpm to separate and remove larger particles' deposits. The supernatant then was dialyzed against deionized water for 3 days by a dialysis tube of 5 kDa to remove excess unreacted reagents.

### Characterization

Phase analysis was done with a Philips BW3710 XRD machine using Cu-Kα radiation (λ = 0.154 nm), step size of 0.02°, and time per step of 0.5 s. FTIR spectrum was recorded on a Perkin Elmer, Spectrum 400 Fourier transform infrared spectrometer. The Atomic Force Microscopy (AFM) image was taken by a Park Scientific Instruments, CP, Auto probe AFM. For AFM analysis, the diluted CQDs solution was sonicated in an ultrasonic bath and then a drop was brought on a cleaved clean mica surface. By evaporation of the solvent, the CQDs were left on the mica surface. The Gd content of the CQDs was determined by ICP-OES Varian 730-ES. The optical properties of the synthesized CQDs were studied with the aid of a Perkin Elmer Lambda 25 UV–Vis spectrophotometer and a Varian Cary Eclipse PL spectrophotometer. The PL spectroscopy was performed at different excitation wavelengths.

The quantum yield (QY) was calculated using quinine sulfate in 0.1 M H_2_SO_4_ with a refractive index of 1.33 and a QY of 54% as the reference with excitation at 365 nm. The QY was calculated by the integrated PL intensity which is the area under the PL curve over the excitation wavelength range. The QY was determined through the following equation:

1 $${\text{QY}} = {\text{QY}}_{{\text{R}}} \left( {{\text{I}}/{\text{I}}_{{\text{R}}} } \right)\left( {{\text{A}}_{{\text{R}}} /{\text{A}}} \right)\left( {\eta /\eta_{{\text{R}}} } \right)^{{2}}$$

where the R subscript refers to the reference material, A is the absorbance at the excitation wavelength, η is the refractive index of solvent (that η/η_R_ was considered 1), and I is the integrated emission intensity^12^.

### Fluorescence imaging

For fluorescence imaging of the synthesized CQDs, an inverted microscope (Nikon, TE-2000) was applied. Mouse fibroblasts (Mouse C_34_/connective tissue-L929) were provided from the National Cell Bank of Iran, Pasteur Institute. First, the cells were cultured in an RPMI-1640 medium containing 50 units of penicillin, 50 µg streptomycin in each mL of the culture medium, and 10% FBS in an incubator at 37 °C with 5% CO_2_ and 85% humidity. After 4 days, the cells were detached by digestion with trypsin (0.25%) for 1 min at room temperature and were prepared in suspensions with 4 × 10^4^ cells/mL. In each well of a 4 wells plate, 500 µl of the cell suspension was added and placed in an incubator for 24 h. Then, 1% and 3% of the synthesized CQDs-containing solution were added to the cells and incubated for 24 h. The samples then were characterized with the inverted microscope.

### Relaxometric measurements

The relaxation times (T1 and T2) of the synthesized Gd-doped CQDs were measured by varying concentrations using a clinical 3 T MRI scanner (Siemens, MAGNETOM Prisma). T1-weighted magnetic resonance imaging was performed under repetition time/echo time (TR/TE) = 750/7.4 ms, matrix size = 256 × 256, slice thickness 2.0 mm, and field of view = 25 × 25 cm^2^. Longitudinal relaxation rates were recorded with variable TR (100–10,000 ms) values and static TE (7.4 ms) values.

## Results and discussions

### Characterization of the synthesized CQDs

**Figure 1:**
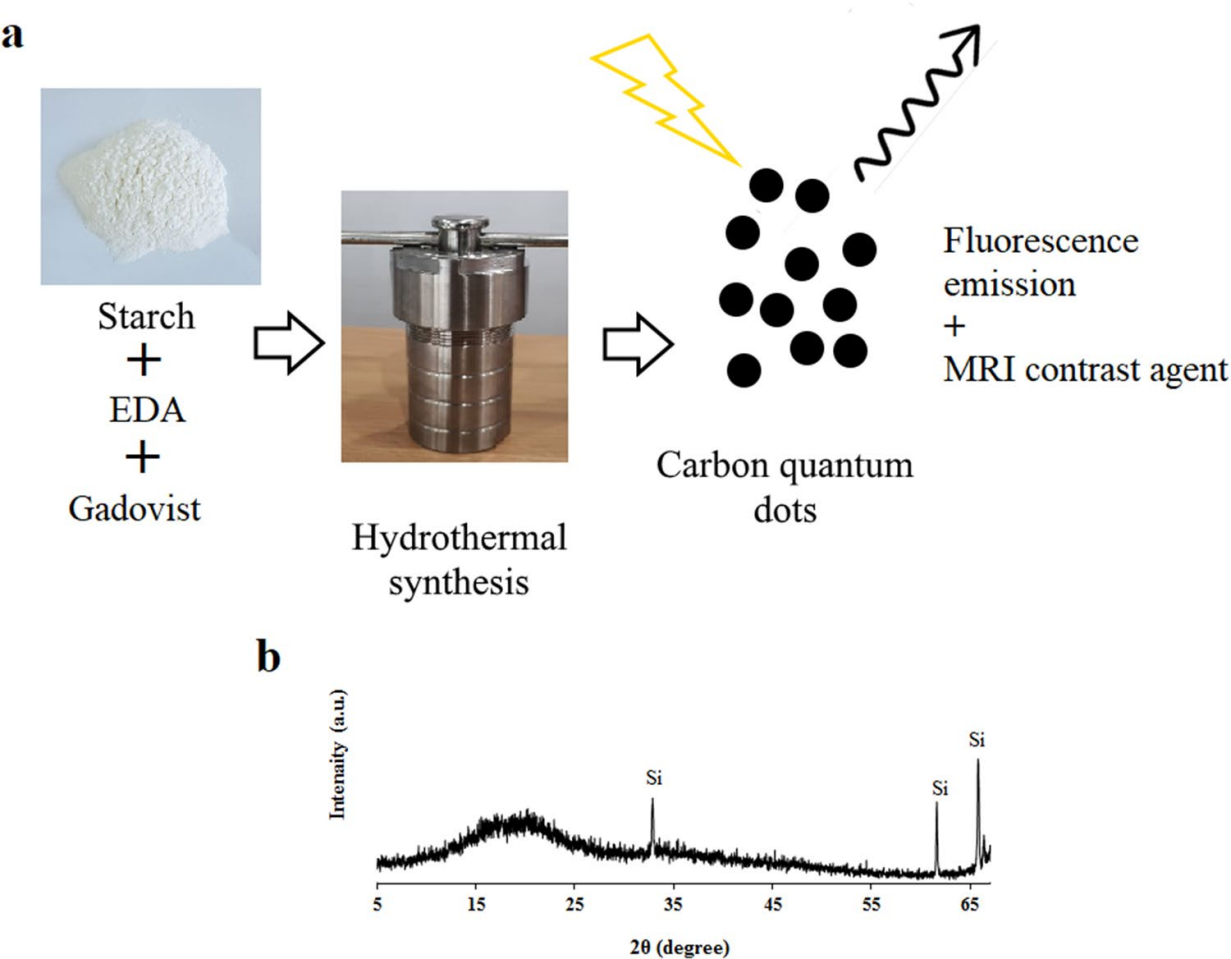
(**a**) The synthesizing method of the Gd-doped CQDs from starch as the carbon source, (**b**) diffraction pattern of the synthesized CQDs on a Si substrate.

Figure 1 shows the synthesizing method and the diffraction pattern of the synthesized CQDs. Ignoring the Si substrate peaks, the hydrothermally treated sample consists of a broad peak centered at 2θ = 18.9° (002) which is evidence of highly amorphous carbon^44^.

**Figure 2:**
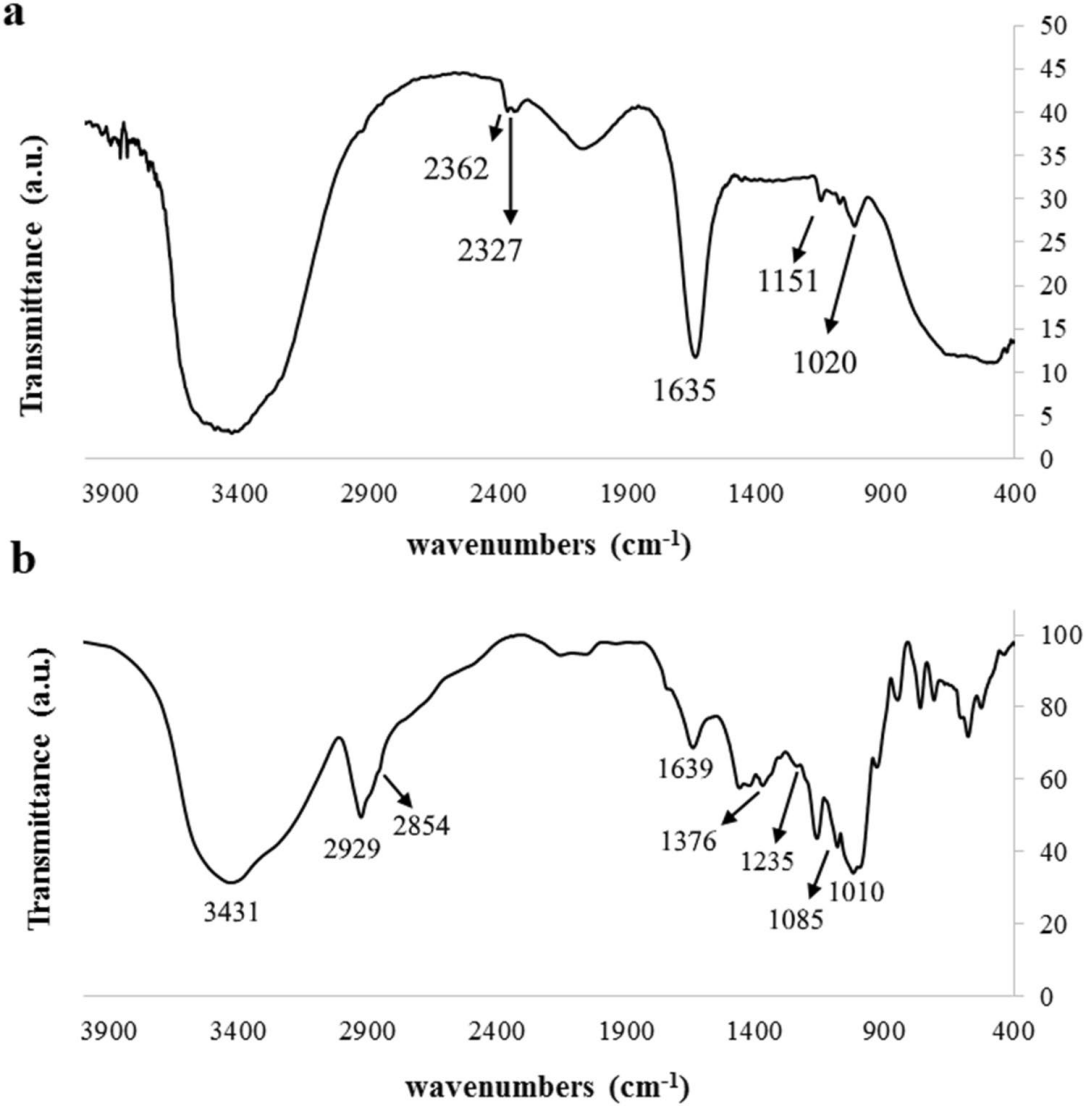
(**a**) The FTIR spectrum of the (**a**) starting starch and (**b**) the synthesized CQDs.

The FTIR spectrum of the starch (starting precursor) and the synthesized CQDs is illustrated in Fig. 2. The peaks in the FTIR spectrum of the starch are solely related to the pure starch while the FTIR spectrum of the synthesized CQDs exhibits peaks at 3431 and 2929 cm^−1^ which correspond to –OH and C–H groups stretching and bending vibrations, respectively. These peaks are attributed to the presence of carbohydrates in the starch. The peak at 1235 cm^−1^ shows the presence of the stretching –C–O– group. Peaks at 1085 and 1639 cm^−1^ correspond to the existence of the –C–O–C– and –C = C– groups. The 1376 cm^−1^ peak belongs to symmetric carboxylate stretching which is evidence of oxidizing of the aldoses/ketoses during the carbonization process^45^. The peak at 3431 cm-^−1^ is attributed to the absorption bands of O–H and N–H stretching vibrations of amine^46^. The peak at 2854 cm^−1^ corresponds to C–H stretching vibration and the peak at 1421 cm^−1^ corresponds to C–N stretching vibration^46,47^.

**Figure 3:**
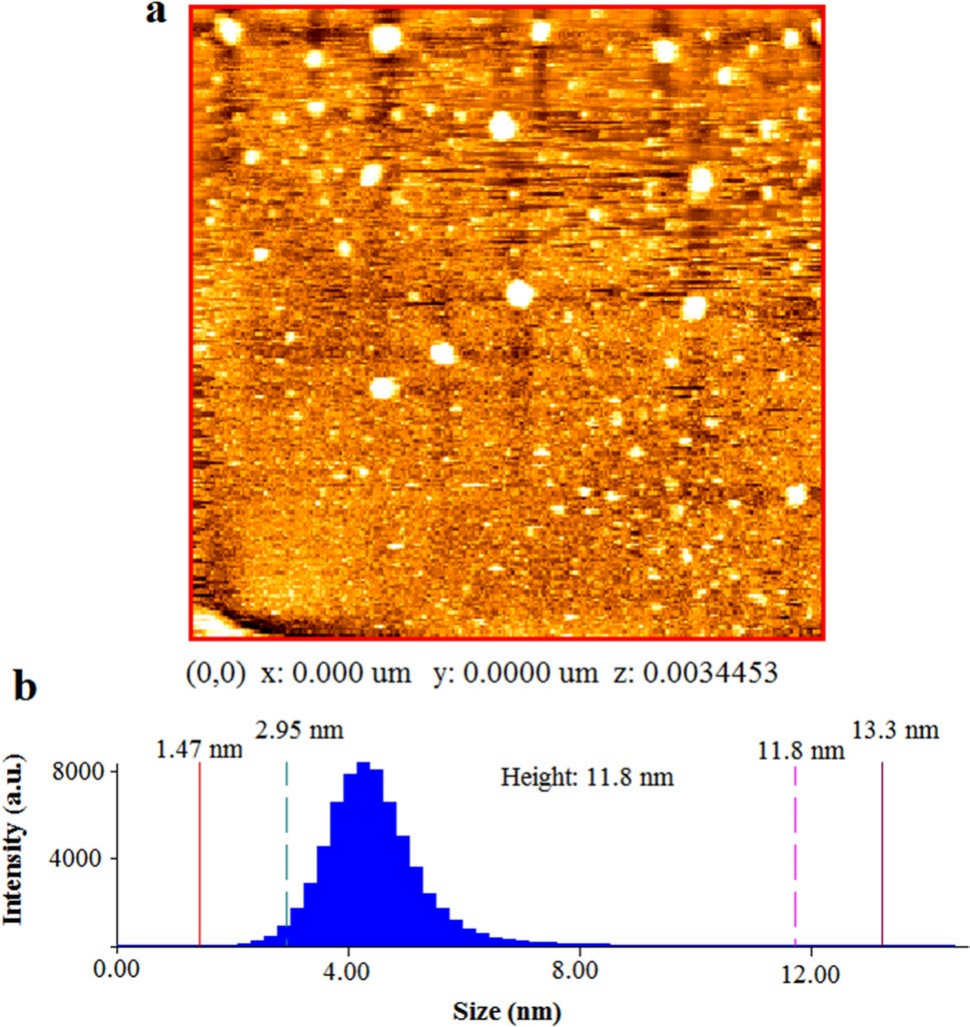
The AFM results of the synthesized CQDs; (**a**) AFM image of the CQDs on a cleaved mica surface, (**b**) size distribution histogram of the CQDs.

Figure 3a represents the AFM image of the CQDs which are dispersed on a mica substrate. The image shows that CQDs have a height of 3 nm. Figure 3b is the related histogram of the size distribution of the CQDs. The histogram indicates that most of the CQDs have a size of 3 nm, while all of them fall below 8 nm.

**Figure 4:**
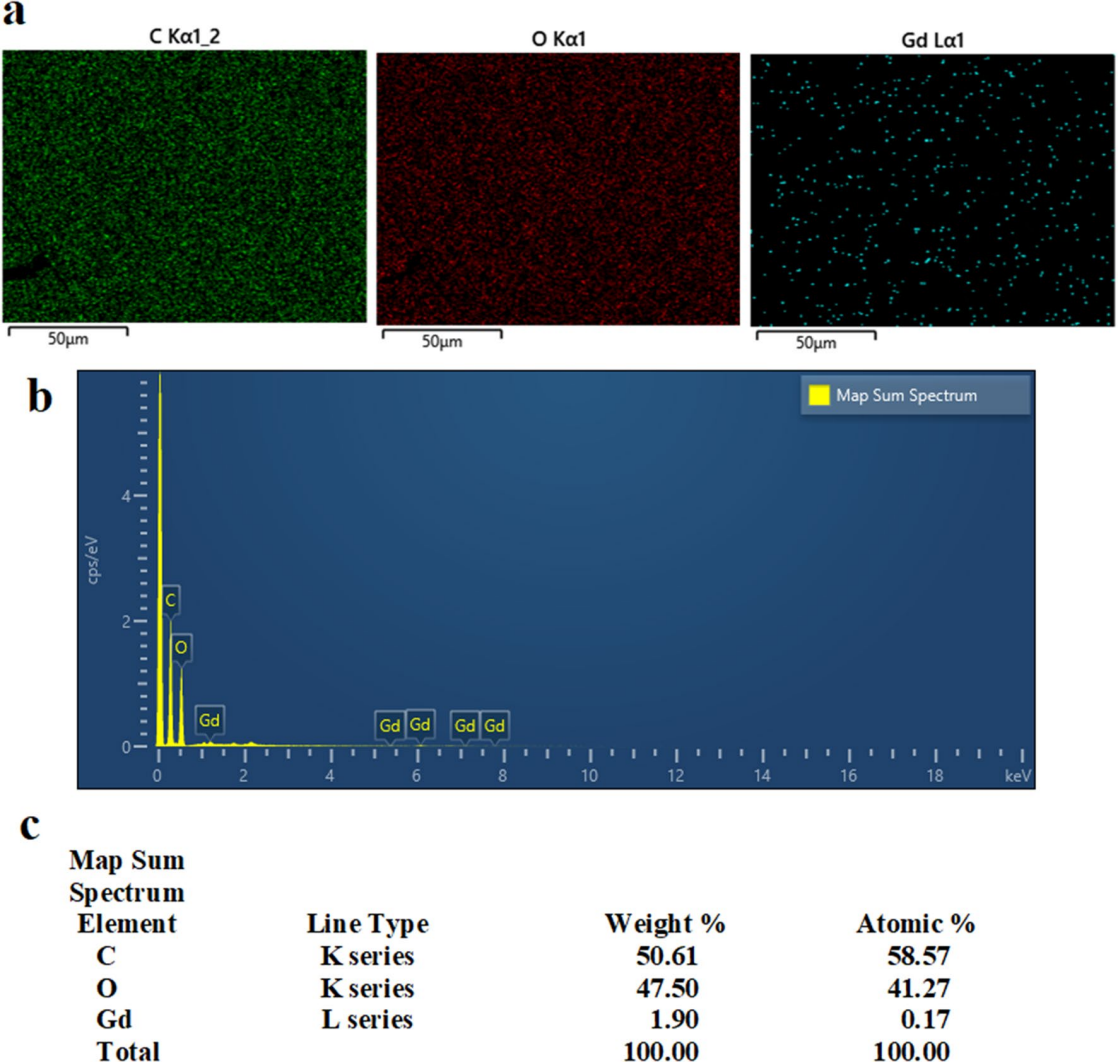
EDS analysis of the synthesized CQDs, (**a**) map of C, O, and Gd, (**b**) EDS map sum spectrum of the elements in the CQDs sample, (**c**) weight and atomic percent of the elements in the CQDs.

Figure 4 shows the EDS chemical analysis of the synthesized sample. The results of the EDS confirm the presence of Gd in the synthesized CQDs which might be in the form of chelates or doped atoms or ions. The sample mainly consists of C and O while the contribution of H has been neglected due to the less accuracy in the detection of H in the EDS measurements. Oxygen might belong to surface functional groups. The ICP-OES characterization also showed the content of the Gd (in the form of chelates, ions, or doped atoms) in the synthesized CQDs was 706 ppm.

### Optical properties

**Figure 5:**
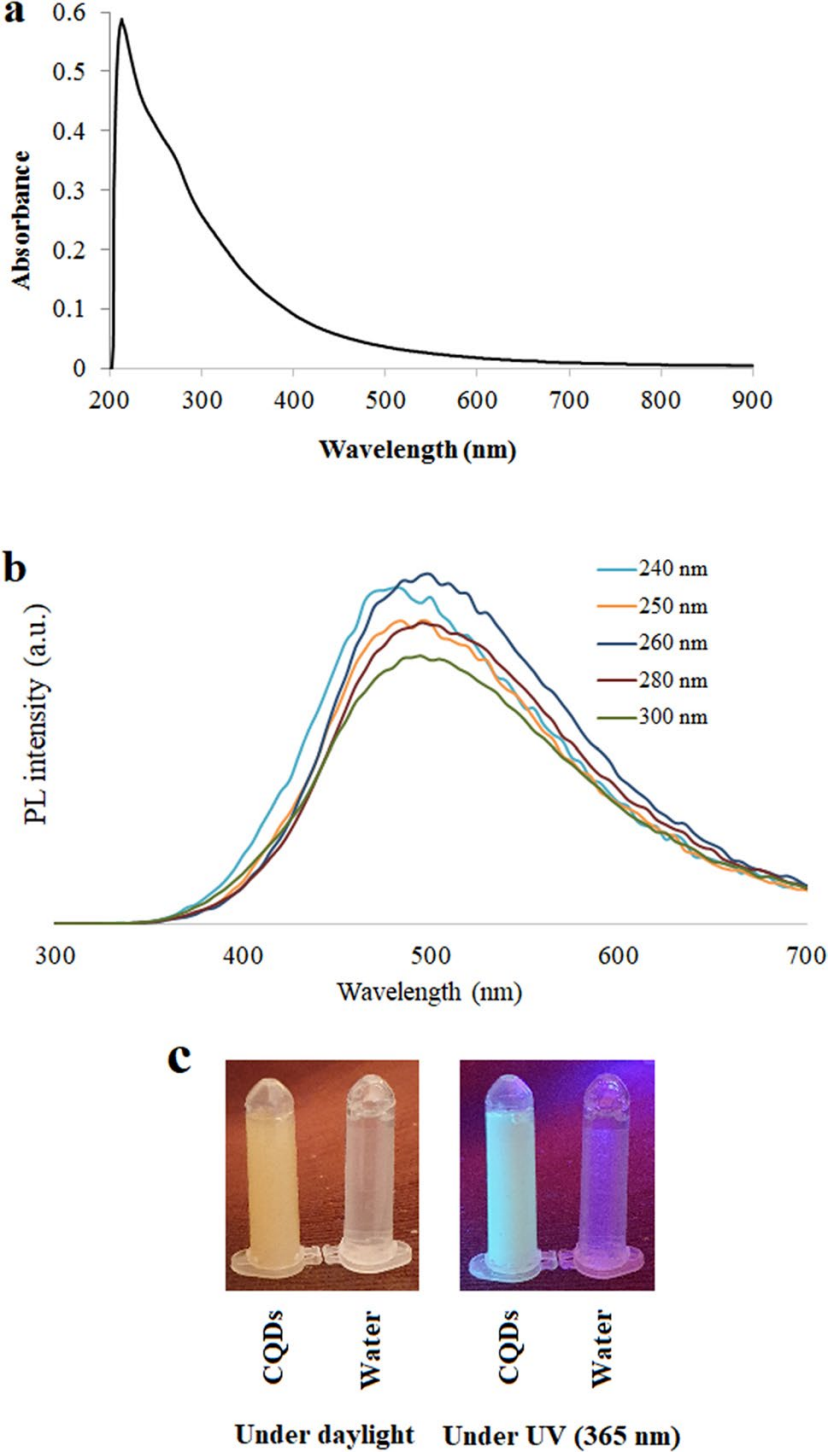
(**a**) The UV–Vis absorption spectrum of the CQDs solution with a concentration of 0.2 mg/mL. (**b**) PL spectroscopy spectra of the synthesized CQDs at different excitation wavelengths. (**c**) The CQDs in comparison with deionized water under UV and visible light.

The UV–Vis absorption spectrum of the CQDs solution (0.2 mg/mL) is shown in Fig. 5a. It can be seen that the synthesized CQDs have absorption in the UV region tailing up to 600 nm. The observed UV absorption peak occurred at 271 nm. The UV absorption peak implies that the synthesized CQDs can be excited with a wavelength in the UV region.

Figure 5b is the PL spectroscopy spectra of the synthesized CQDs with different excitation wavelengths and Fig. 5c is the CQDs solution under daylight and UV irradiation compared to pure water. It can be seen that by excitation at different wavelengths, spectra with different maximum emission intensity wavelength is generated. Figure 5b shows that the emission peak wavelength is dependent on the excitation wavelength. The excitation wavelength-dependent PL behavior of CQDs has been reported before^48–50^. The excitation dependence of the PL emission might be due to the presence of the CQDs with different sizes, surface chemistry, and different emissive traps^51^. The strongest emission from CQDs has usually been observed in the deep blue or blue regions of the spectrum and this emission is a result of radiative recombination of electron–hole pairs or quantum size effect (intrinsic state emission). The strong emission that can be seen in the green region of the spectrum in some cases arises from surface defects emission^52^. Since the emission in the synthesized sample is in the green region, it might be the surface defects that are dominant in the PL emission. The QY of the synthesized CQDs was calculated to be 13.2%.

The application of amino groups has a substantial effect on the synthesis and fluorescence characteristics of the CQDs. In graphene quantum dots (GQDs) of the same size, by increasing the number of amine groups, the emission wavelength increased. Furthermore, because of the reduction of the epoxide and carboxylic groups that act as centers for non-radiative electron–hole pair recombination the QY increased. Because of strong interaction with –NH_2_ groups, the primary amines at the edges of the GQDs have higher HOMO (highest occupied molecular orbital) than hydrogen-terminated groups. The resonance between molecule orbitals in the -NH_2_ groups and delocalized π orbital leads to the optical band gap narrowing^51^.

**Figure 6:**
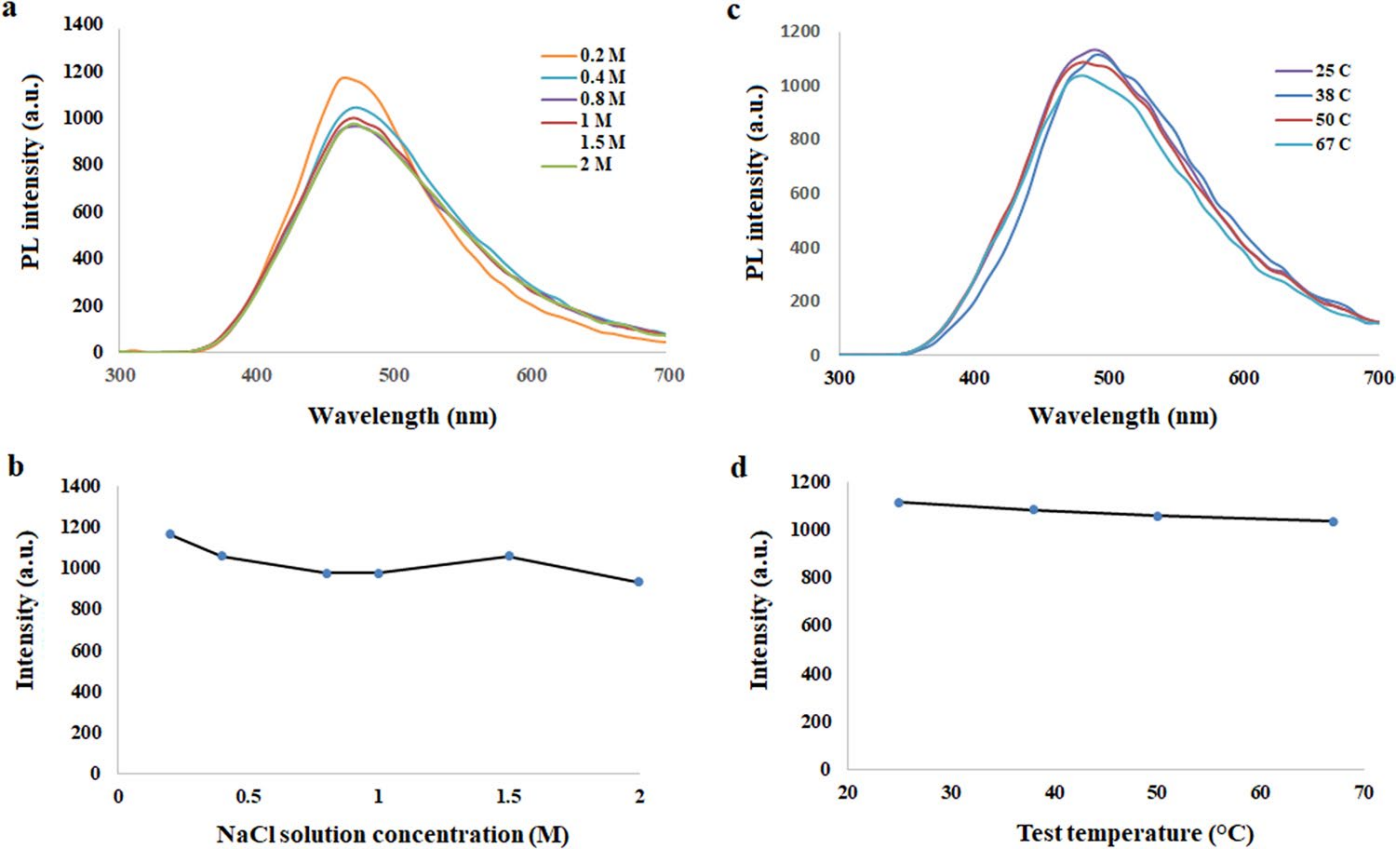
(**a**) The PL spectra of the CQDs in the ionic solutions with different molarity and (**b**) the diagram showing maximum observed PL intensity in different ionic solutions by excitation at 300 nm. (**c**) PL spectra at different temperatures and (**d**) the diagram of maximum observed intensity at different temperatures by excitation at 300 nm.

The synthesized CQDs can sustain their PL in ionic conditions or higher temperatures. Sustained fluorescence emission in ionic solutions with different ionic strengths is important for the application of these dots in the human body environment since the salt concentration is 0.3 M in the human body. This was studied by the excitation of the CQDs with a wavelength of 300 nm and measuring their fluorescence intensity emission. It can be seen in Fig. 6a,b that the fluorescence intensity of the CQDs in NaCl solutions ranging from 0.2 to 2 M is almost constant. The CQDs can maintain their photostability in salt solutions as well.

The effect of temperature on the fluorescence emission intensity of CQDs can be observed in Fig. 6c,d. Since the CQDs for biomedical applications are expected to maintain their emission in the human body environment, their fluorescence emission intensity at different temperatures was investigated by measuring the fluorescence emission by excitation at 300 mm. The results confirmed that the CQDs can still show sustained PL at the human body temperature which is beneficial for biomedical applications.

### In vitro fluorescence imaging

**Figure 7:**
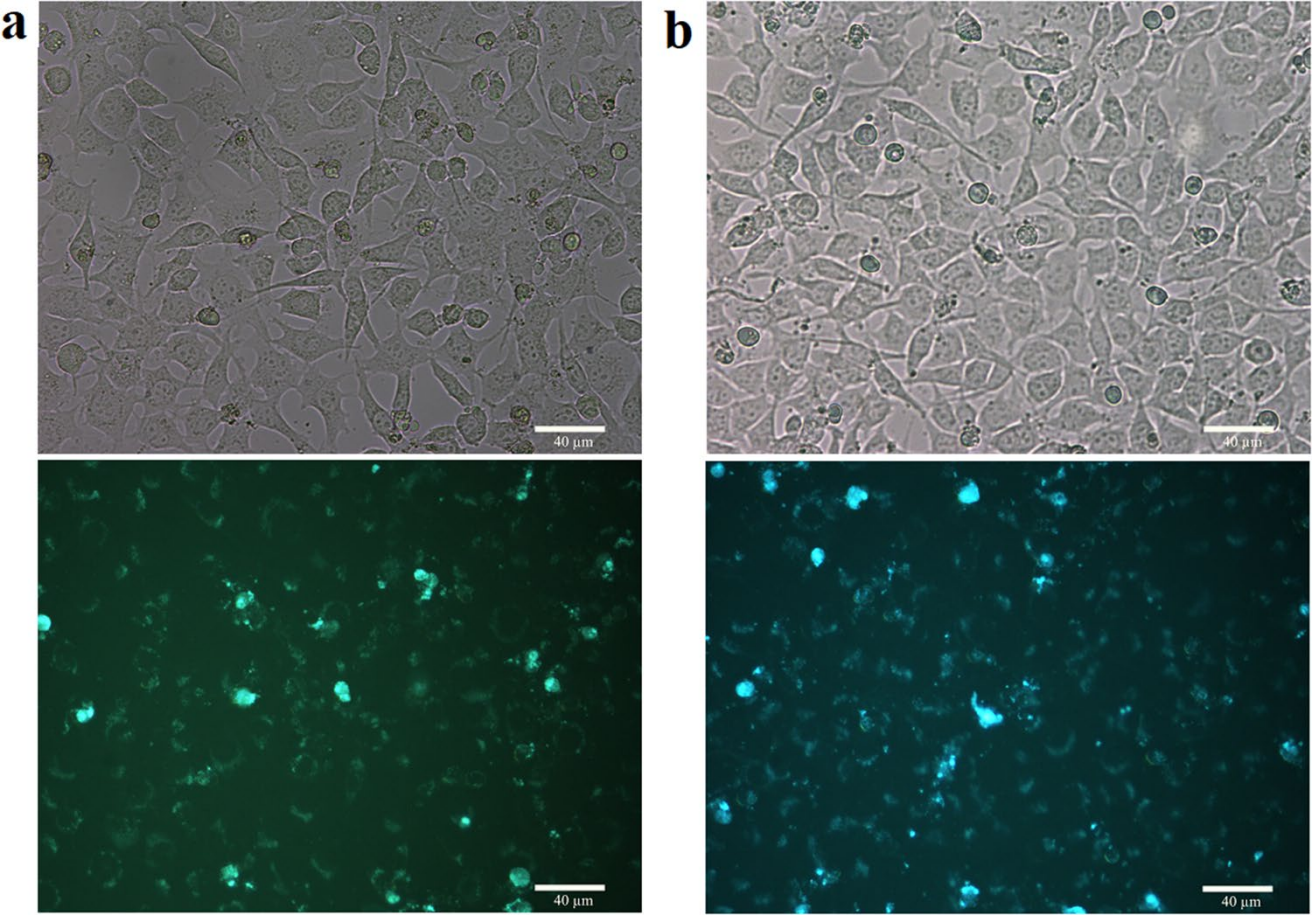
Fluorescence microscopy images of the Mouse C34/connective tissue-L929 cells with CQDs as the fluorescent probes; bright-field images on the top and fluorescent images on the bottom for (**a**) 1% and (**b**) 3% of the CQDs.

Due to the strong PL, water-solubility, and facile synthesis of the CQDs from the natural ingredient it is worthy to investigate if they can be used in fluorescence bioimaging. The in vitro bioimaging of the CQDs with different concentrations of the solution was studied by their incubation with mouse fibroblasts (Mouse C34/connective tissue-L929) cells. In a series of experiments, 1% and 3% of the synthesized CQDs were incubated with the cells. The results of fluorescence imaging of the incubated cells are illustrated in Fig. 7. It can be observed that the CQDs could be distributed over the cytoplasm, while they were not able to penetrate the cell nucleus. These results are in agreement with previous reports that have claimed CQDs can stain both cell membrane and cytoplasm but rarely can be internalized into the cell nucleus^53^. The CQDs could be internalized into the cells by endocytosis^37^. The cell images reveal that they have reserved their living morphology during the incubation and imaging analysis, indicating low damage of the synthesized CQDs to the incubated cells. Since all the cells are not stained by the CQDs, it seems that the synthesized CQDs had been able to penetrate partly into the cells.

### Magnetic resonance imaging

**Figure 8:**
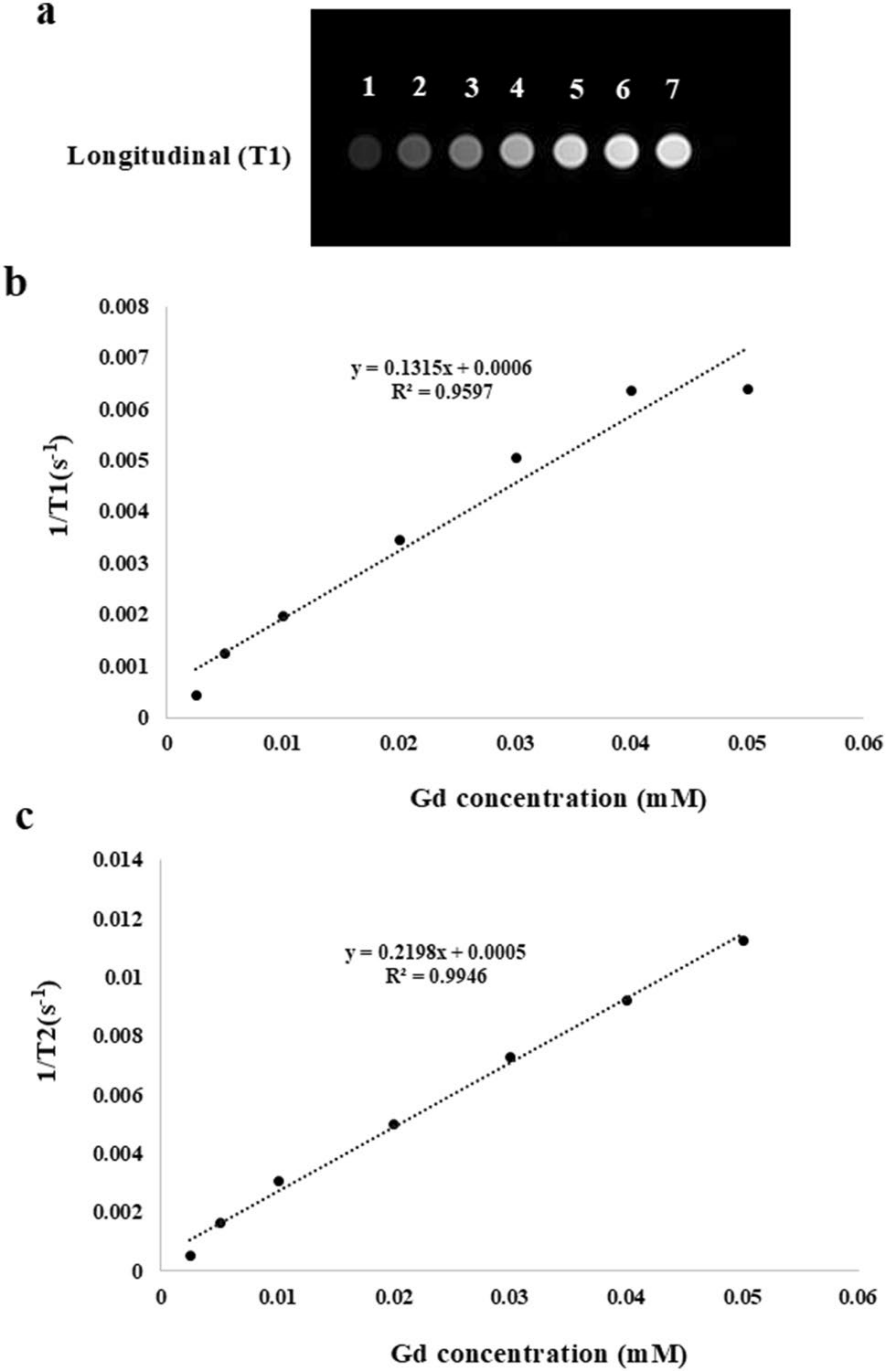
(**a**) T1-weighted magnetic resonance phantom images of Gd-doped CQDs; the samples 1–7 correspond to the Gd concentrations of 0.0025, 0.005, 0.01, 0.02, 0.03, 0.04, 0.05 mM, respectively. (**b**) The linear relationship between r1 and Gd concentration, (**c**) Linear relationship between transverse relaxivities (r2) and Gd concentration.

The magnetic relaxation behavior of the synthesized Gd-doped CQDs was measured to investigate the application of the dots as MRI contrast agents. The magnetic relaxation of the Gd-doped CQDs was evaluated by the preparation of samples with different dilutions. Figure 8a shows the T1-weighted MR images at various Gd concentrations. It can be seen that the Gd-doped CQDs induced a positive contrast that brightens by increasing the Gd concentration in the samples. The specific relaxivities (r1 and r2) of the samples were calculated by determining the slope of the linear plot of longitudinal and transverse relaxation rates (1/T1 and 1/T2) vs. the concentration of the Gd. The longitudinal relaxivity value (r1) was found to be 0.1315 mM^−1^ s^−1^ (Fig. 8b) which is lower than Gd-DOTA as the commercial contrast agent which is 4.5 ± 0.3 mM^−1^ s^−1^^54^. The transverse relaxivity (r2) of the Gd-doped CQDs can be derived from Fig. 8c which is the plot of transverse relaxation rates (1/T2) vs. the concentration of the Gd. The transverse relaxivity for the Gd-doped CQDs was 0.2198 mM^−1^ s^−1^. The direct interactions between Gd ions and hydrogen protons will affect the r1 value^38^. For the MRI contrast agents that are based on the particles, the utilization of nanoparticles with small sizes is important for achieving enhanced relaxivities. The Gd-doped CQDs with small size and high specific surface ratios would lead to an increase in the dipole–dipole interactions between hydrogen protons and Gd ions which increases the r1 value^40^.

The magnetic resonance characteristics of the contrast agent depend on the relaxivity ratio (r2/r1). If r2/r1 ≥ 10 the contrast agent behaves as the T2-weighted contrast agent and if r2/r1 < 2 the material would act as a T1-weighted contrast agent^55^. The relaxivity ratio for the Gd-doped CQDs is 1.66 which means they can be considered as T1-weighted contrast agents.

## Conclusions

CQDs were synthesized by an easy hydrothermal process of starch, ethylenediamine, and Gd ions-containing contrast agent. The synthesized CQDs with a size distribution of 2–8 nm showed excitation-wavelength PL emission and UV absorption peak at 271 nm. The CQDs had sustained PL in ionic solutions with different ionic strengths as well as different temperatures up to 65 °C. The fluorescence microscopy of the cells that had been incubated with Gd-doped CQDs confirmed that the dots could be well distributed over the cytoplasm, while they were not able to penetrate the cell nucleus. The MRI of the Gd-doped CQDs showed an extremely high longitudinal relaxivity value of 218.28 mM^−1^ s^−1^. Therefore they can be used as efficient T1 contrast agents in lower dosages. The synthesized CQDs can be used for simultaneous fluorescence imaging and MRI. The easy synthesis, multifunctionality, and high relaxivity value of these CQDs are promising for their utilization in diagnosis biomedical applications.

## Data Availability

The datasets used and/or analyzed during the current study are available from the corresponding author upon reasonable request.
